# Case Report: Fibrin-associated large B-cell lymphoma developing within a cystic lesion of the adrenal gland: unexpected guest

**DOI:** 10.3389/fimmu.2026.1787193

**Published:** 2026-05-08

**Authors:** Haneen Al-Maghrabi, Bayan Ali Alghamdi

**Affiliations:** 1 Department of Pathology and Laboratory Medicine, King Faisal Specialist Hospital and Research Center, Jeddah, Saudi Arabia; 2 Department of Pathology, King Abdulaziz University Hospital, Jeddah, Saudi Arabia

**Keywords:** adrenal gland, endocrine, Epstein–Barr virus, fibrin-associated large B-cell lymphoma, lymphoma

## Abstract

Fibrin-associated large B-cell lymphoma (FA-LBCL) is recognized as a distinct entity in the 5th edition of the World Health Organization (WHO) classification of hematolymphoid tumors. It is a rare Epstein–Barr virus (EBV)-positive B-cell neoplasm that develops in areas of chronic fibrin deposition, associated with (pseudo)cystic cavities, long-standing hematomas, myxomas, or prosthetic devices. Cystic lymphangiomatous lesions of the adrenal gland are uncommon. Benign lymphovascular abnormalities are typically asymptomatic, but can be detected incidentally and removed to exclude other conditions. This report describes an exceptionally rare case involving a 42-year-old woman diagnosed incidentally with FA-LBCL arising within cystic lymphangiomatous lesions of the adrenal gland. We present a detailed account of the cytomorphological, immunohistochemical, and molecular characteristics of this FA-LBCL case and provide a concise overview of this emerging lymphoma subtype.

## Introduction

Fibrin-associated large B-cell lymphoma (FA-LBCL) is a rare Epstein–Barr virus (EBV)-associated lymphoma that has been recognized as a distinct entity in the 5th edition of the World Health Organization (WHO) classification of hematolymphoid tumors (1). It was previously classified as a variant of diffuse large B-cell lymphoma (DLBCL) arising in the setting of chronic inflammation (2). FA-LBCL typically develops in the context of long-standing inflammatory processes and occurs within fibrinous material located in confined natural or acquired anatomic spaces most commonly within cardiac myxomas, vascular thrombi (3), pseudocysts (4), hematomas (5), and various prosthetic devices (6), including breast implants (7). This lymphoma is often diagnosed incidentally in surgical resection for non-neoplastic reasons and generally does not form a discrete mass, instead appearing as sparse microscopic clusters of malignant cells, which exhibit a high proliferative index, but show no evidence of tissue invasion. Because of its rarity, minimal tumor burden, and wide spectrum of presentations, FA-LBCL can be diagnostically challenging, particularly when it manifests as a largely necrotic cystic lesion. The existing literature on this entity is limited, with only case reports and small case series available. These lymphomas typically display a non-germinal center immunophenotype and are associated with EBV infection. They generally show a more favorable prognosis compared with conventional DLBCL and DLBCL arising in the context of chronic inflammation (5). Here, we describe a case of FA-LBCL involving the adrenal gland presenting as cystic lesions along with a literature review.

## Clinical presentation

**Figure 1:**
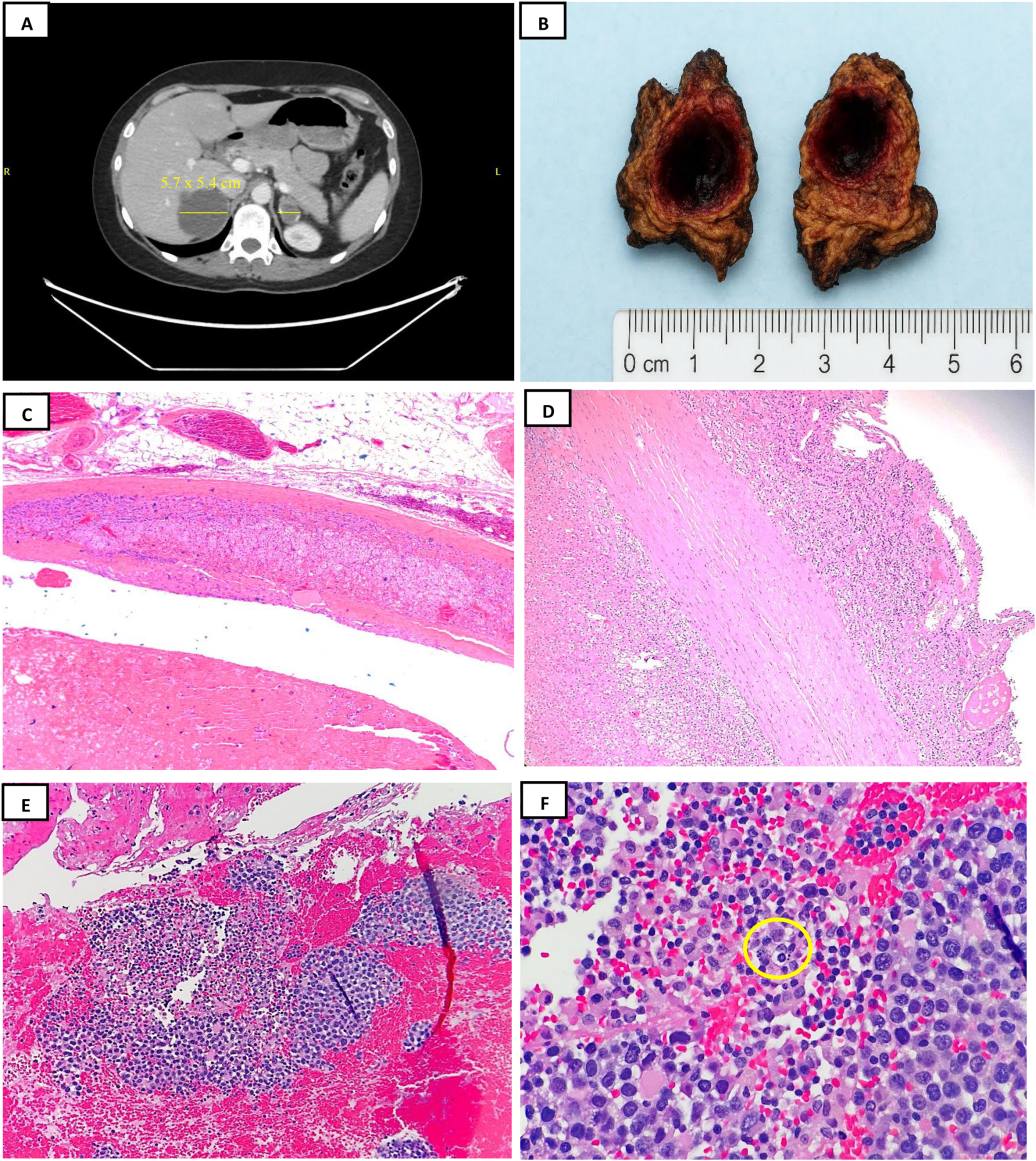
Radiology, gross, and histopathology examination with hematoxylin and eosin (H&E) stain. **(A)** Contrast-enhanced CT of the pelvis demonstrated bilateral non-enhancing adrenal lesions, with minimally complex cysts. A slight increase in size on the *right* and a decrease on the *left side*. No evidence of malignancy was identified in the abdomen or pelvis. **(B)** Adrenal cystic lesion with a smooth tan outer surface containing cystic tan-brown area filled with hemorrhagic material. **(C)** Thick fibrous wall surrounding the pseudocyst, and residual adrenal gland tissue is present at the periphery. Importantly, this adrenal tissue contains no tumor cells (H&E, ×4). **(D)** Thick fibrous wall with chronic inflammation, blood, and foamy histiocytes (note the lining of the cyst) (H&E, ×10). **(E)** Inside the hemorrhagic material are small clusters of neoplastic cells floating in fibrin and pink amorphous acellular eosinophilic material (H&E, ×20). **(F)** The neoplastic cells are large, pleomorphic, and hyperchromatic, with irregular nuclear membranes and prominent nucleoli. Note the apoptotic debris and mitotic figure (circle) (H&E, ×40).

A 42-year-old woman with no previous medical history presented to our institution with episodes of hypotension, associated with palpitation, tremor, and paleness. She had no history of hypertension or cerebrovascular disease. There was no sweating, muscle spasm, purple stria, fever, or night sweat. At the time the patient presented to our institution, complete blood count (CBC) showed a white blood cell (WBC) count of 4.17K/μl (normal range, 3.90–11), but the differential WBC count was all within normal limits. The adrenal gland hormonal status was normal. Computed tomography (CT) scan showed a right adrenal fluid attenuation lesion measuring 5.7 cm × 5.4 cm compared with the 4.7 cm × 4.6 cm with peripheral calcification in a CT done 6 months ago. The overall findings represented a minimally complex cyst (**Figure 1A**). In addition, a left adrenal lesion measured 2.1 cm × 2.1 cm, previously 3.5 cm × 2.5 cm with no definite post-contrast enhancement. The patient was subsequently accepted as a case of bilateral non-functioning adrenal incidentaloma, with a negative aldosterone–renin ratio test, and was admitted for right laparoscopic adrenalectomy.

## Pathologic findings

**Figure 2:**
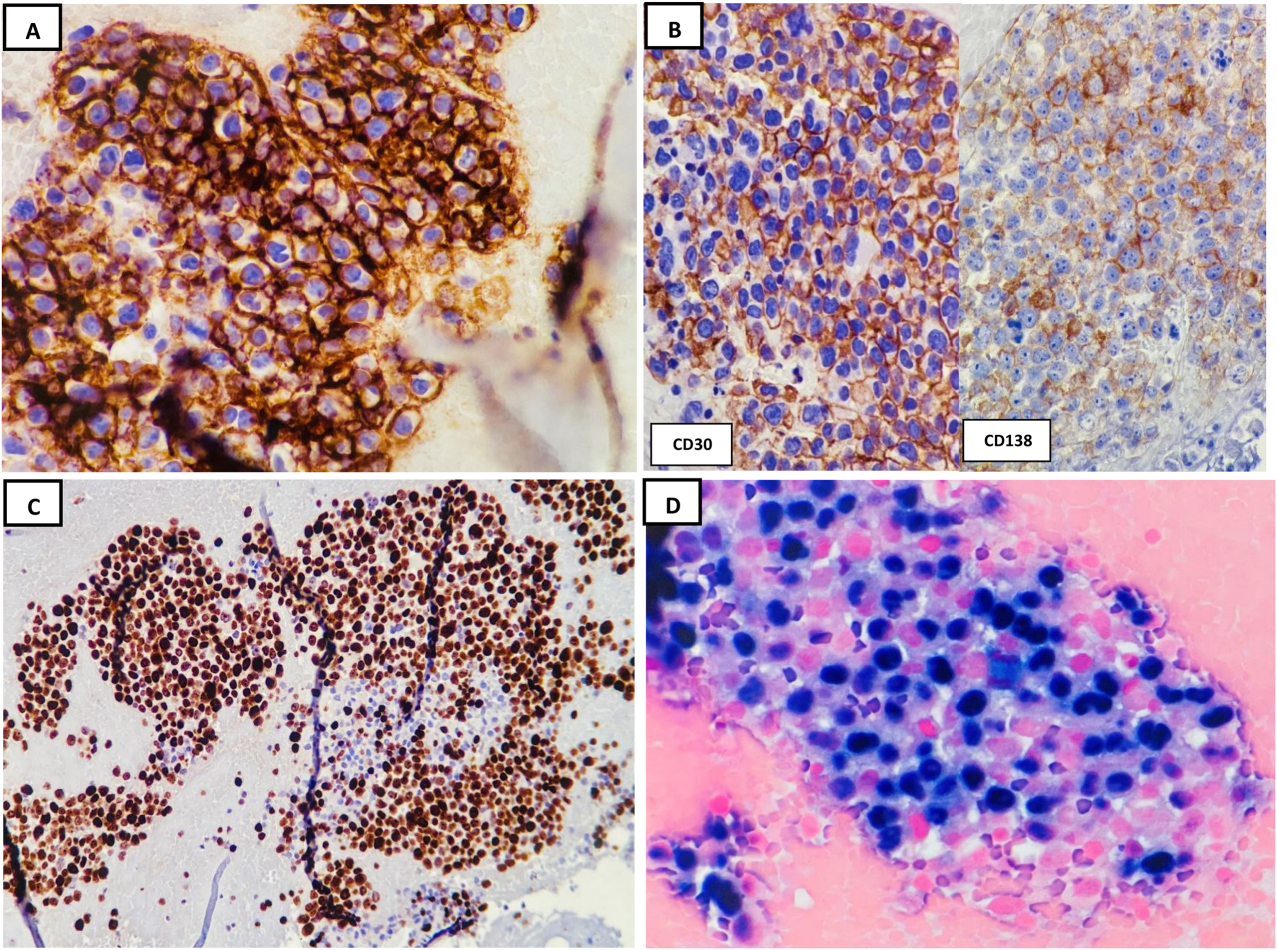
**(A)** Immunohistochemical analysis performed on the neoplastic cells revealed positive staining for CD20. **(B)** CD30 (bright and strong membranous expression) and CD138 (lighter and subset expression) (×40). **(C)** Ki-67 proliferative index revealed >90% of the neoplastic cells (×20). **(D)** *In situ* hybridization for Epstein–Barr virus (EBV) (EBER) showed positive staining virtually in all neoplastic cells (×40).

Gross examination revealed an 18.8 g cystic mass, which measured 5.7 cm in the largest dimension. The outer surface of the cyst was smooth with dense fibro-fatty adhesions. The inner surface of the cyst contained soft, brown, hemorrhagic material (**Figure 1B**) located adjacent to the remaining adrenal gland tissue. No gross evidence of tumor was identified. Microscopic examination showed a thick, fibrous wall with focal calcifications, containing clusters of adrenal cortical cells, hemosiderin-laden cells, and occasional small lymphocytes. The lumen of the pseudocyst largely lacked an epithelial lining, although there were occasional spindle-shaped cells in a few areas, possibly endothelial in origin. Some histologic sections demonstrated small foci of chronic inflammatory cells outlining the outer surface of the pseudocyst wall (**Figures 1C, D**). The pseudocyst contents consisted of abundant acellular fibrinous or amorphous eosinophilic material, hemorrhage, necrotic debris, hemosiderin-laden macrophages, few lymphocytes, and scattered clusters of large neoplastic cells. These neoplastic cells were predominantly floating in the acellular fluid near the fibrous wall, arranged in clusters, ribbons, and single cells (**Figure 1E**). The neoplastic cells exhibited large nuclei with irregular nuclear membranes, coarse chromatin, prominent nucleoli, and amphophilic cytoplasm. Rare cells showed plasmacytoid-like differentiation. Mitotic figures and apoptotic bodies were present (**Figure 1F**). There was no lymphomatous infiltration of adjacent adrenal and cystic tissues. A panel of immunohistochemical analysis done with working controls showed target atypical lymphoid cells positive for CD45, CD20 (**Figure 2A**), PAX5, CD79a, CD30 (**Figure 2B**), BCL2, and MUM1/IRF4. A subset of cells partially expressed CD38 and CD138, although the neoplastic cells lacked lymphoplasmacytic differentiation (**Figure 2B**). Neoplastic cells were negative for CD3, CD5, CD10, CD56, BCL6, CyclinD1, HHV8, and ALK1. The Ki-67 index showed a 90% proliferative rate (**Figure 2C**). *In situ* hybridization–EBV-encoded small RNA (EBER) was positive in all neoplastic cells (**Figure 2D**). Molecular testing of B-cell monoclonality by immunoglobulin heavy chain (IGH) gene rearrangement could not be conducted due to the insufficient quantity of cellular DNA. The diagnosis of adrenal FA-LBCL was based on morphologic and immunophenotypic grounds. LBCL foci were present in 10 out of 12 histologic sections analyzed.

## Clinical outcomes

**Figure 3:**
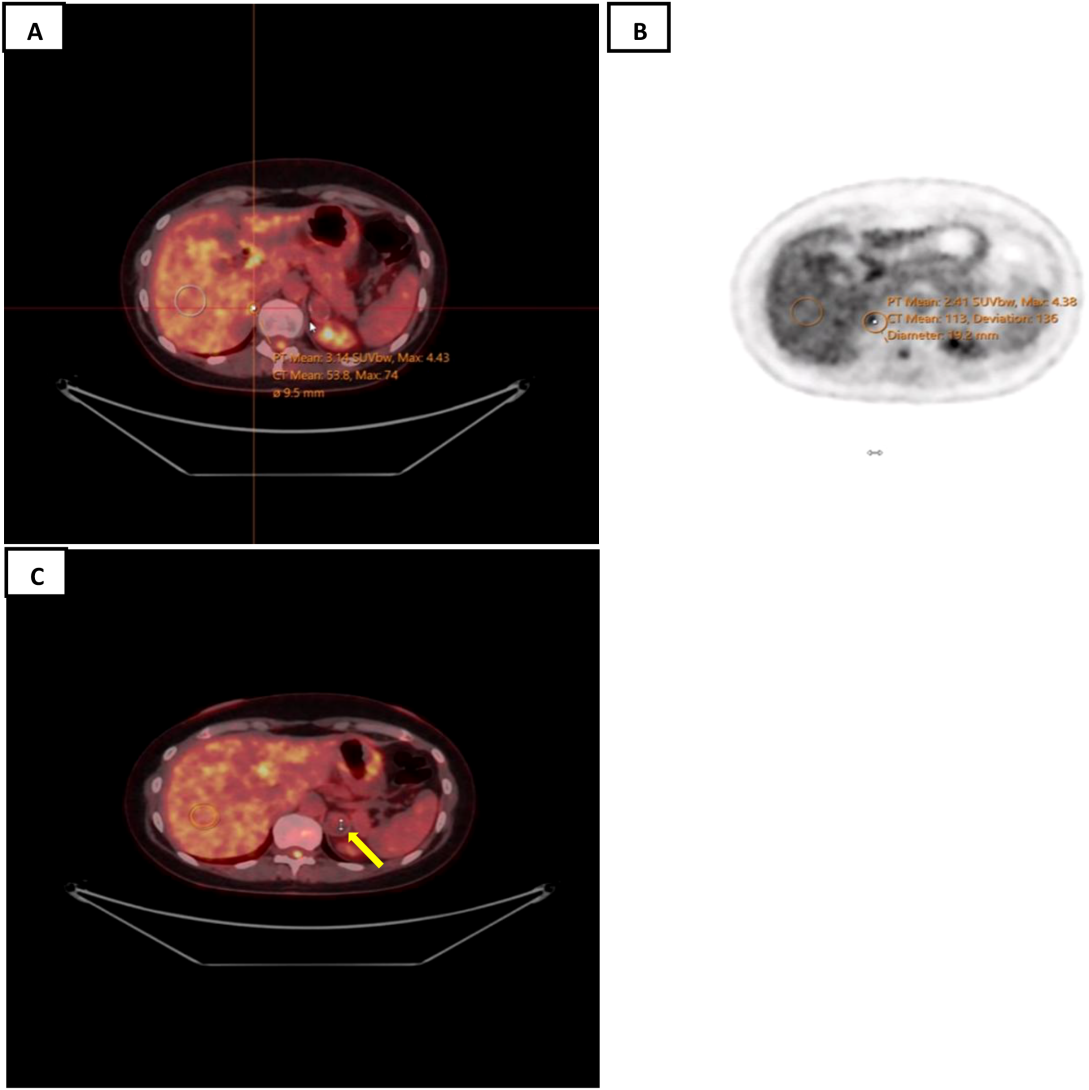
**(A, B)** Whole body ^18^F-FDG PET/CT scan showed suspicious mild focal activity within the non-enlarged right adrenal gland with tiny calcification. **(C)** Non-FDG-avid peripherally calcified lesion in the left adrenal gland measuring up to 2.3 cm (*yellow arrow*).

Staging studies using fluorodeoxyglucose (^18^F-FDG) positron emission tomography (PET) combined with a CT scan were performed as per standard protocol from the cranial vertex down to the mid-thigh level, which showed no significant FDG uptake within the lesion involving the left adrenal gland, measuring 2.3 cm × 2.1 cm with peripheral calcification. Tiny calcification in the right adrenal gland with possible mild focal activity [standardized uptake value (SUV) = 4.0] was observed (**Figure 3**). There were no suspicious hypermetabolic lymph nodes above or below the diaphragm and no suspicious hypermetabolic splenic or bone marrow lesions. Due to suspected residual disease, the multidisciplinary tumor board (MDT) recommended treatment with six cycles of rituximab, cyclophosphamide, doxorubicin, vincristine, and prednisone (R-CHOP). The patient is well and disease-free 12 months after diagnosis.

## Discussion

FA-LBCL is an uncommon type of EBV-driven large B-cell lymphoma that is confined to a specific anatomic location or that develops within acquired spaces. It is currently acknowledged as a distinct EBV-driven neoplasm in the 5th edition of the WHO classification of hematolymphoid tumors (1). It is a rare entity, with roughly 60 cases reported in the English-language literature (2–9). It is typically diagnosed incidentally in histological sections taken for other conditions, similarly with the present case. The most frequent locations for FA-LBCL are pseudocysts occurring at various anatomical sites. FA-LBCL can also arise in other fluid-filled structures, such as hematomas, cardiac atrial myxomas, and hyperplastic thyroid nodules. In addition, it may involve prosthetic materials, including endovascular grafts, prosthetic heart valves, and other metallic implants (2, 8). Overall, primary lymphomas of the adrenal gland account for roughly 1% of cases (9). To date, adrenal gland FA-LBCL has been reported in only five cases (2, 3, 8–10) (**Table 1**). The morphologic features of FA-LBCL resemble those described in other reported cases. The neoplasm consists of aggregates/clusters of atypical large cells of centroblastic or immunoblastic appearance, situated within fibrinous or amorphous eosinophilic material along with degenerating cellular debris. Neoplastic cells commonly show moderate nuclear pleomorphism with irregular nuclear contours and prominent nucleoli, along with numerous mitotic figures and frequent apoptotic bodies (11). These lesions typically show little or no chronic inflammatory infiltrate. In pseudocyst cases, a band-like collection of lymphocytes and/or plasma cells may be present along the outer layer of the fibrous wall (12). FA-LBCL typically does not show infiltration into the adjacent tissue parenchyma, effacement of nearby normal structures, or true mass forming (10, 11). Immunohistochemistry analysis of these neoplastic cells showed pan-B-cell marker expression, including CD19, CD20, CD79a, and PAX5, while lacking T-cell markers. Nearly all reported cases show positivity for MUM1/IRF4 and negativity for CD10, indicating a non-germinal center B-cell phenotype. BCL2 expression is common, and CD30 and programmed death-ligand 1 (PDL1) may be expressed (8), while Ki-67 generally demonstrates a high proliferative index in viable tumor cells (3, 8). The proportion of MYC-positive cells varies between 15% and 50%, with a median of 20% in tumor cells (8). The expression of p53 in the majority of cases ranges from low to moderate (3, 8, 9). It is worth noting that a very rare case reported in the literature was diagnosed as FA-LBCL with lymphoplasmacytic differentiation, characterized by its B-cell origin with loss of CD20 expression (5).

**Table 1:** Clinical features of patients with fibrin-associated large B-cell lymphoma (FA-LBCL) presenting in the adrenal gland.

	Reference	Sex	Age (years)	Clinical presentation	Size (cm)	Treatment	Outcome
1	Boroumand et al. (2)	Female	63	Right upper quadrant abdominal pain for 6 months	26	6 cycles R-CHOP, followed by radiation therapy	Free of disease after 40 months
2	Boyer et al. (8)	Male	70	Bladder outlet obstruction	7	Surgical resection only	Free of disease after 14 months
3	Zanelli et al. (3)	Female	71	Lower limb edema and marked abdominal distension	34	NA	NA
4	Jia et al. (9)	Female	36	Early-onset hypertension	6.9	4 cycles R-CHOP + two doses of rituximab	Free of disease after 37 months
5	Kircher et al. (10)	Female	45	Long-standing (for 10 years) slowly increasing mass	6.5	Complete resection+ local irradiation	Free of disease after 12 months
6	Our present case	Female	42	hypotension	5.7	6 cycles R-CHOP	Free of disease after 12 months

R-CHOP, rituximab, cyclophosphamide, doxorubicin, vincristine, and prednisone; *NA*, not available.

EBV infection is detected in the majority of FA-LBCL cases; however, exceptionally uncommon EBV-negative cases have been described (13). The virus typically exhibits an EBV latency III infection pattern, which is characterized by a strong, diffuse expression of EBV nuclear antigen 2 (EBNA2) (3). EBV latent membrane protein 1 (LMP-1) is also strongly expressed, whereas *Bam*HI Z fragment leftward open reading frame 1 (BZLF-1) is detected in none or only a very small number of cells (3, 9, 10). EBV-negative FA-LBCL is extremely uncommon, and its underlying mechanisms remain unclear, although it is likely driven by a distinct pathogenic process. In one case described by Baugh et al. (13), the tumor cells exhibited a germinal center B-cell phenotype according to the Hans algorithm and was also EBV-negative, further indicating a potentially distinct pathogenesis. Molecular analyses have been conducted in a limited number of FA-LBCL cases. In the majority of cases where testing was carried out, clonal immunoglobulin rearrangements were detected, which did not exhibit rearrangements of MYC, BCL2, or BCL6 (14). The underlying mechanisms of tumor pathogenesis are poorly understood. It has been proposed that FA-LBCL develops in anatomical locations that are low in oxygen and difficult for the host immune system to reach. Consequently, these sites are immunologically secluded and cannot be reached by the EBV-specific T cells of the host. Under these conditions, cells latently infected with EBV can reactivate the virus, leading to the expansion of EBV-infected B cells. These B cells may subsequently develop into clonal populations, as evidenced by the clonal immunoglobulin gene rearrangements observed in the majority of cases where testing was conducted (15). Nonetheless, these EBV-driven clonal B-cell proliferations do not exhibit other oncogenic gene rearrangements, such as those involving MYC, BCL2, or BCL6 (3, 8). The pathogenic contribution of chronic inflammation to the development of these tumors remains unclear. It is possible that chronic inflammation may not be strictly necessary. Instead, the confined environment in which FA-LBCL develops could be sufficient to shield the tumors from cytolytic T-cell activity, leading to localized immunodeficiency. A number of authors have proposed that the expression of hypoxia-inducible factor-1 alpha (HIF-1α) indicates the presence of hypoxia, which could also occur in a confined space, thereby supporting this notion. Hypoxic conditions and oncogenic signaling facilitate tumor progression by enhancing cell survival, angiogenesis, and drug resistance. Consequently, elevated HIF-1α levels serve as a metabolic regulator and a clinical marker for aggressive disease and unfavorable outcomes (16). Moreover, cell lines from prolonged chronic inflammation can release interleukin-10 (IL-10), a cytokine recognized for its ability to suppress immune responses by inhibiting T-cell proliferation (2, 17). In addition, interleukin-6 (IL-6), produced by pro-inflammatory cytokines, can promote the proliferation of EBV-positive B cells and potentially contribute to the disease pathogenesis. Within a confined environment, prolonged exposure to IL-6 may promote the survival of EBV-infected B cells and help them evade T-cell-mediated immune surveillance (18).

The findings reported in this case are consistent with this theory. The cystic degeneration of the adrenal gland may be attributed to its large size and impaired vascular supply. It remains uncertain whether hormonal imbalance contributes to the cystic changes. FA-LBCL generally carries a favorable prognosis and can often be successfully managed through complete surgical removal of the tumor, accompanied by long-term follow-up (12). Surveillance is typically recommended instead of systemic chemotherapy after surgery (19). In the present case, the abnormal findings on the postoperative PET/CT scan led to the administration of R-CHOP chemotherapy. At the most recent follow-up, the patient remained free of lymphoma.

The differential diagnosis of FA-LBCL involving the adrenal gland includes benign and malignant neoplasms such as endothelial or epithelial cysts, pseudocysts, EBV-positive DLBCL, DLBCL associated with chronic inflammation, and EBV-positive T-cell lymphoproliferative disorder (20). Endothelial cysts are benign cysts lined by a flattened, bland endothelium. However, the endothelial cell lining may be difficult to visualize in some cases. Revascularization and papillary endothelial hyperplasia are often present. Epithelial cysts are a similar type of cysts lined by a single layer of bland epithelium. Epithelial cells are cuboidal, hobnailed to flattened, and can mimic the endothelial lining. Pseudocysts usually contain no lining, which requires a careful search for intact lining that may reveal the endothelium, reclassifying a pseudocyst as an endothelial cyst. The cystic contents include hemorrhage, debris, and calcification (21). EBV-positive DLBCLs are clonal large B-cell lymphomas associated with EBV infection occurring in patients without a prior history of immunodeficiency. They most commonly affect older individuals, show a male predominance, and frequently present at an advanced stage. The tumors consist of large, transformed cells with variable morphology, appearing either in sheets or within a polymorphous inflammatory background. The involved tissues typically demonstrate diffuse architectural effacement, and areas of geographic necrosis and angioinvasion may also be seen (22). DLBCL associated with chronic inflammation is a type of large B-cell lymphoma that develops within long-standing inflamed tissue and is linked to EBV infection. The majority of cases occur in individuals with a history of more than 20 years of pyothorax, typically following artificial pneumothorax treatment for pulmonary tuberculosis or tuberculous pleuritis. The disease usually originates in the pleura or pleural space and may extend to adjacent structures. It is an aggressive lymphoma with a poor prognosis (8, 23). A rare case of EBV-positive T-cell lymphoproliferative disorder that developed in a chronic pericardial hematoma has been reported. This may represent the T-cell counterpart of FA-LBCL and could easily be distinguished through careful morphologic, immunophenotypic, and molecular studies (20).

## Conclusion

FA-LBCL is a rare subtype of EBV-associated lymphoma that is now recognized as a distinct entity in the 5th edition of the WHO classification of hematolymphoid neoplasms. We describe the clinicopathologic features of a patient with adrenal gland FA-LBCL, diagnosed after presenting with cystic mass on imaging. The diagnosis of FA-LBCL can be challenging, particularly when it appears as a markedly necrotic cystic lesion, and its evaluation often requires proper grossing and multidisciplinary collaboration for appropriate management.

## Data Availability

Written informed consent was obtained from the individual(s) for the publication of any potentially identifiable images or data included in this article.
